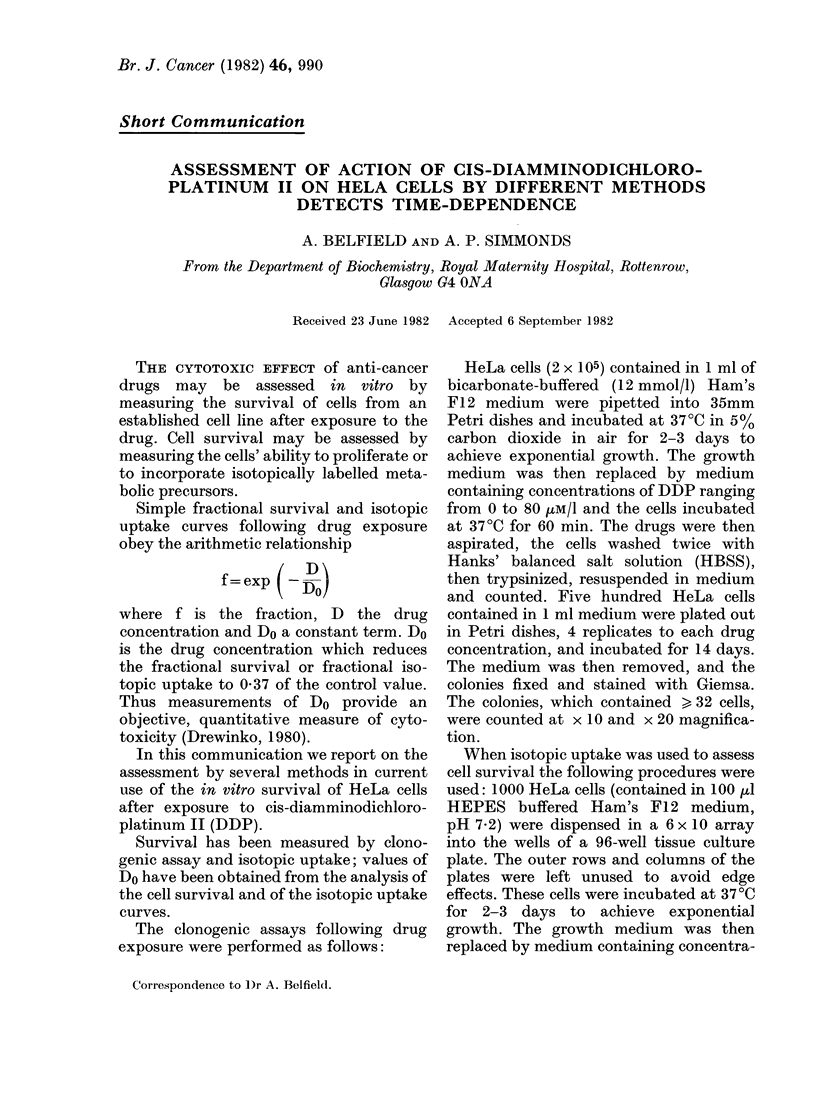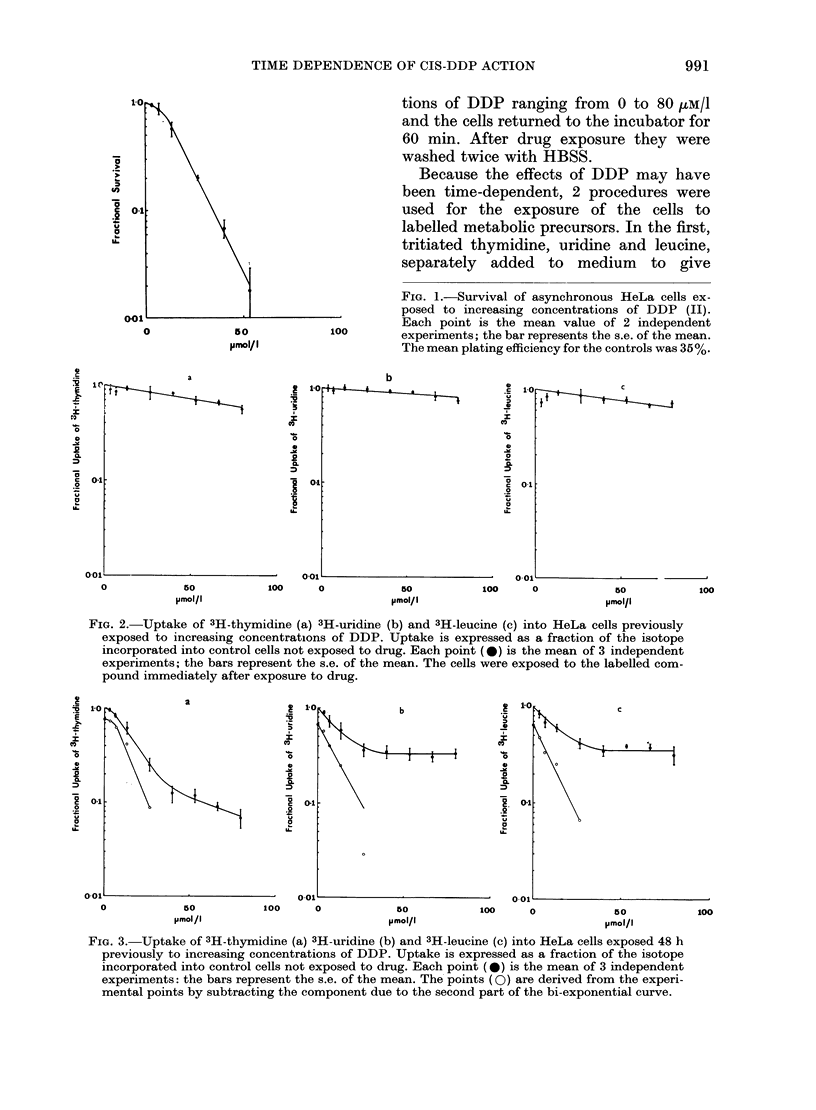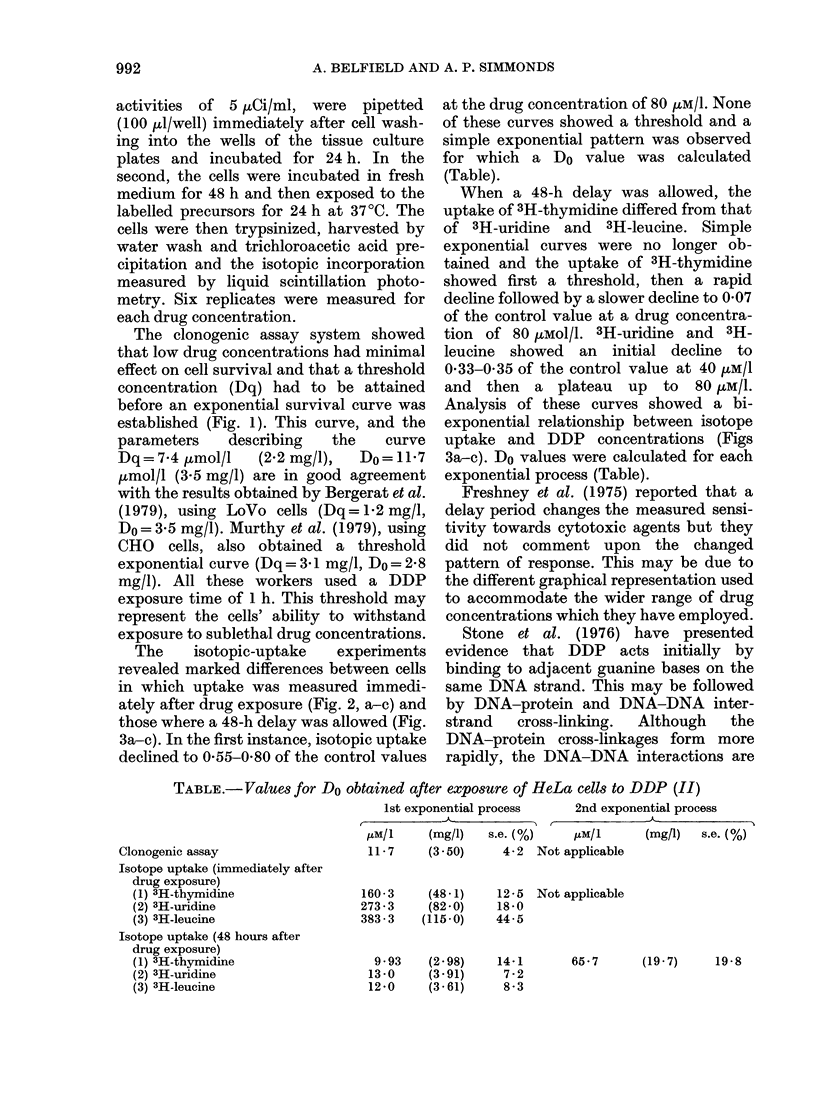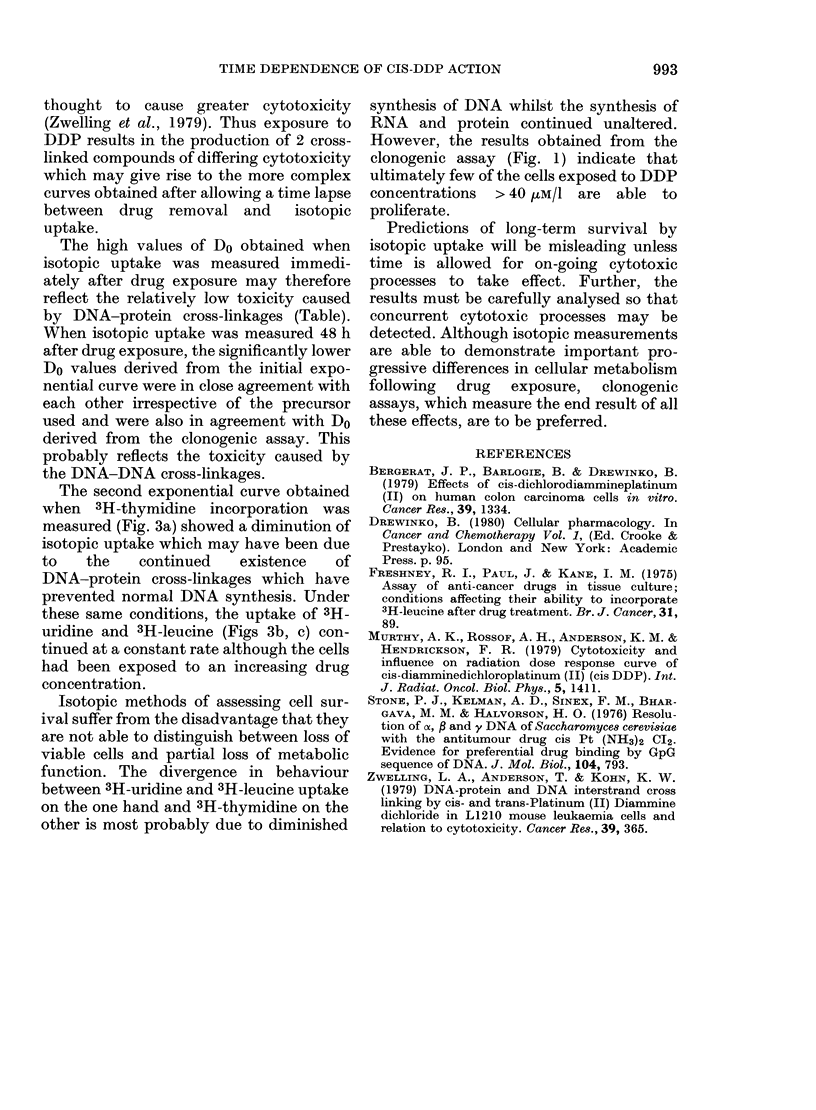# Assessment of action of cis-diamminodichloro-platinum II on HeLa cells by different methods detects time-dependence.

**DOI:** 10.1038/bjc.1982.314

**Published:** 1982-12

**Authors:** A. Belfield, A. P. Simmonds


*Br. J. Cancer* (1982) **46**, 990

***Short Communication***

### ASSESSMENT OF ACTION OF CIS-DIAMMINODICHLORO-PLATINUM II ON HELA CELLS BY DIFFERENT METHODS DETECTS TIME-DEPENDENCE

A. BELFIELD AND A. P. SIMMONDS

*From the Department of Biochemistry, Royal Maternity Hospital, Rottenrow, Glasgow G4 0NA*

Received 23 June 1982 Accepted 6 September 1982

THE CYTOTOXIC EFFECT of anti-cancer drugs may be assessed *in vitro* by measuring the survival of cells from an established cell line after exposure to the drug. Cell survival may be assessed by measuring the cells' ability to proliferate or to incorporate isotopically labelled metabolic precursors.

Simple fractional survival and isotopic uptake curves following drug exposure obey the arithmetic relationship

$$f = \exp\left(-\frac{D}{D_0}\right)$$

where f is the fraction, D the drug concentration and $D_0$ a constant term. $D_0$ is the drug concentration which reduces the fractional survival or fractional isotopic uptake to 0·37 of the control value. Thus measurements of $D_0$ provide an objective, quantitative measure of cytotoxicity (Drewinko, 1980).

In this communication we report on the assessment by several methods in current use of the *in vitro* survival of HeLa cells after exposure to cis-diamminodichloroplatinum II (DDP).

Survival has been measured by clonogenic assay and isotopic uptake; values of $D_0$ have been obtained from the analysis of the cell survival and of the isotopic uptake curves.

The clonogenic assays following drug exposure were performed as follows:

HeLa cells ($2 \times 10^5$) contained in 1 ml of bicarbonate-buffered (12 mmol/l) Ham's F12 medium were pipetted into 35mm Petri dishes and incubated at 37°C in 5% carbon dioxide in air for 2–3 days to achieve exponential growth. The growth medium was then replaced by medium containing concentrations of DDP ranging from 0 to 80 $\mu$M/l and the cells incubated at 37°C for 60 min. The drugs were then aspirated, the cells washed twice with Hanks' balanced salt solution (HBSS), then trypsinized, resuspended in medium and counted. Five hundred HeLa cells contained in 1 ml medium were plated out in Petri dishes, 4 replicates to each drug concentration, and incubated for 14 days. The medium was then removed, and the colonies fixed and stained with Giemsa. The colonies, which contained $\geqslant 32$ cells, were counted at $\times 10$ and $\times 20$ magnification.

When isotopic uptake was used to assess cell survival the following procedures were used: 1000 HeLa cells (contained in 100 $\mu$l HEPES buffered Ham's F12 medium, pH 7·2) were dispensed in a $6 \times 10$ array into the wells of a 96-well tissue culture plate. The outer rows and columns of the plates were left unused to avoid edge effects. These cells were incubated at 37°C for 2–3 days to achieve exponential growth. The growth medium was then replaced by medium containing concentra-

Correspondence to Dr A. Belfield.

tions of DDP ranging from 0 to 80 $\mu$M/l and the cells returned to the incubator for 60 min. After drug exposure they were washed twice with HBSS.

Because the effects of DDP may have been time-dependent, 2 procedures were used for the exposure of the cells to labelled metabolic precursors. In the first, tritiated thymidine, uridine and leucine, separately added to medium to give

FIG. 1.—Survival of asynchronous HeLa cells exposed to increasing concentrations of DDP (II). Each point is the mean value of 2 independent experiments; the bar represents the s.e. of the mean. The mean plating efficiency for the controls was 35%.

FIG. 2.—Uptake of $^3$H-thymidine (a) $^3$H-uridine (b) and $^3$H-leucine (c) into HeLa cells previously exposed to increasing concentrations of DDP. Uptake is expressed as a fraction of the isotope incorporated into control cells not exposed to drug. Each point (●) is the mean of 3 independent experiments; the bars represent the s.e. of the mean. The cells were exposed to the labelled compound immediately after exposure to drug.

FIG. 3.—Uptake of $^3$H-thymidine (a) $^3$H-uridine (b) and $^3$H-leucine (c) into HeLa cells exposed 48 h previously to increasing concentrations of DDP. Uptake is expressed as a fraction of the isotope incorporated into control cells not exposed to drug. Each point (●) is the mean of 3 independent experiments: the bars represent the s.e. of the mean. The points (○) are derived from the experimental points by subtracting the component due to the second part of the bi-exponential curve.

activities of 5 μCi/ml, were pipetted (100 μl/well) immediately after cell washing into the wells of the tissue culture plates and incubated for 24 h. In the second, the cells were incubated in fresh medium for 48 h and then exposed to the labelled precursors for 24 h at 37°C. The cells were then trypsinized, harvested by water wash and trichloroacetic acid precipitation and the isotopic incorporation measured by liquid scintillation photometry. Six replicates were measured for each drug concentration.

The clonogenic assay system showed that low drug concentrations had minimal effect on cell survival and that a threshold concentration (Dq) had to be attained before an exponential survival curve was established (Fig. 1). This curve, and the parameters describing the curve Dq = 7·4 μmol/l (2·2 mg/l), $D_0$ = 11·7 μmol/l (3·5 mg/l) are in good agreement with the results obtained by Bergerat *et al.* (1979), using LoVo cells (Dq = 1·2 mg/l, $D_0$ = 3·5 mg/l). Murthy *et al.* (1979), using CHO cells, also obtained a threshold exponential curve (Dq = 3·1 mg/l, $D_0$ = 2·8 mg/l). All these workers used a DDP exposure time of 1 h. This threshold may represent the cells' ability to withstand exposure to sublethal drug concentrations.

The isotopic-uptake experiments revealed marked differences between cells in which uptake was measured immediately after drug exposure (Fig. 2, a–c) and those where a 48-h delay was allowed (Fig. 3a–c). In the first instance, isotopic uptake declined to 0·55–0·80 of the control values at the drug concentration of 80 μM/l. None of these curves showed a threshold and a simple exponential pattern was observed for which a $D_0$ value was calculated (Table).

When a 48-h delay was allowed, the uptake of $^3$H-thymidine differed from that of $^3$H-uridine and $^3$H-leucine. Simple exponential curves were no longer obtained and the uptake of $^3$H-thymidine showed first a threshold, then a rapid decline followed by a slower decline to 0·07 of the control value at a drug concentration of 80 μMol/l. $^3$H-uridine and $^3$H-leucine showed an initial decline to 0·33–0·35 of the control value at 40 μM/l and then a plateau up to 80 μM/l. Analysis of these curves showed a bi-exponential relationship between isotope uptake and DDP concentrations (Figs 3a–c). $D_0$ values were calculated for each exponential process (Table).

Freshney *et al.* (1975) reported that a delay period changes the measured sensitivity towards cytotoxic agents but they did not comment upon the changed pattern of response. This may be due to the different graphical representation used to accommodate the wider range of drug concentrations which they have employed.

Stone *et al.* (1976) have presented evidence that DDP acts initially by binding to adjacent guanine bases on the same DNA strand. This may be followed by DNA–protein and DNA–DNA inter-strand cross-linking. Although the DNA–protein cross-linkages form more rapidly, the DNA–DNA interactions are

TABLE.—*Values for $D_0$ obtained after exposure of HeLa cells to DDP (II)*

| | 1st exponential process | | | 2nd exponential process | | |
|---|---|---|---|---|---|---|
| | μM/l | (mg/l) | s.e. (%) | μM/l | (mg/l) | s.e. (%) |
| Clonogenic assay | 11·7 | (3·50) | 4·2 | Not applicable | | |
| Isotope uptake (immediately after drug exposure) | | | | | | |
| (1) $^3$H-thymidine | 160·3 | (48·1) | 12·5 | Not applicable | | |
| (2) $^3$H-uridine | 273·3 | (82·0) | 18·0 | | | |
| (3) $^3$H-leucine | 383·3 | (115·0) | 44·5 | | | |
| Isotope uptake (48 hours after drug exposure) | | | | | | |
| (1) $^3$H-thymidine | 9·93 | (2·98) | 14·1 | 65·7 | (19·7) | 19·8 |
| (2) $^3$H-uridine | 13·0 | (3·91) | 7·2 | | | |
| (3) $^3$H-leucine | 12·0 | (3·61) | 8·3 | | | |

thought to cause greater cytotoxicity (Zwelling *et al.*, 1979). Thus exposure to DDP results in the production of 2 cross-linked compounds of differing cytotoxicity which may give rise to the more complex curves obtained after allowing a time lapse between drug removal and isotopic uptake.

The high values of $D_0$ obtained when isotopic uptake was measured immediately after drug exposure may therefore reflect the relatively low toxicity caused by DNA–protein cross-linkages (Table). When isotopic uptake was measured 48 h after drug exposure, the significantly lower $D_0$ values derived from the initial exponential curve were in close agreement with each other irrespective of the precursor used and were also in agreement with $D_0$ derived from the clonogenic assay. This probably reflects the toxicity caused by the DNA–DNA cross-linkages.

The second exponential curve obtained when $^3$H-thymidine incorporation was measured (Fig. 3a) showed a diminution of isotopic uptake which may have been due to the continued existence of DNA–protein cross-linkages which have prevented normal DNA synthesis. Under these same conditions, the uptake of $^3$H-uridine and $^3$H-leucine (Figs 3b, c) continued at a constant rate although the cells had been exposed to an increasing drug concentration.

Isotopic methods of assessing cell survival suffer from the disadvantage that they are not able to distinguish between loss of viable cells and partial loss of metabolic function. The divergence in behaviour between $^3$H-uridine and $^3$H-leucine uptake on the one hand and $^3$H-thymidine on the other is most probably due to diminished synthesis of DNA whilst the synthesis of RNA and protein continued unaltered. However, the results obtained from the clonogenic assay (Fig. 1) indicate that ultimately few of the cells exposed to DDP concentrations $> 40\ \mu$M/l are able to proliferate.

Predictions of long-term survival by isotopic uptake will be misleading unless time is allowed for on-going cytotoxic processes to take effect. Further, the results must be carefully analysed so that concurrent cytotoxic processes may be detected. Although isotopic measurements are able to demonstrate important progressive differences in cellular metabolism following drug exposure, clonogenic assays, which measure the end result of all these effects, are to be preferred.

### REFERENCES

BERGERAT, J. P., BARLOGIE, B. & DREWINKO, B. (1979) Effects of cis-dichlorodiammineplatinum (II) on human colon carcinoma cells *in vitro*. *Cancer Res.*, **39**, 1334.

DREWINKO, B. (1980) Cellular pharmacology. In *Cancer and Chemotherapy Vol. 1*, (Ed. Crooke & Prestayko). London and New York: Academic Press. p. 95.

FRESHNEY, R. I., PAUL, J. & KANE, I. M. (1975) Assay of anti-cancer drugs in tissue culture; conditions affecting their ability to incorporate $^3$H-leucine after drug treatment. *Br. J. Cancer*, **31**, 89.

MURTHY, A. K., ROSSOF, A. H., ANDERSON, K. M. & HENDRICKSON, F. R. (1979) Cytotoxicity and influence on radiation dose response curve of cis-diamminedichloroplatinum (II) (cis DDP). *Int. J. Radiat. Oncol. Biol. Phys.*, **5**, 1411.

STONE, P. J., KELMAN, A. D., SINEX, F. M., BHARGAVA, M. M. & HALVORSON, H. O. (1976) Resolution of $\alpha$, $\beta$ and $\gamma$ DNA of *Saccharomyces cerevisiae* with the antitumour drug cis Pt $(NH_3)_2$ $CI_2$. Evidence for preferential drug binding by GpG sequence of DNA. *J. Mol. Biol.*, **104**, 793.

ZWELLING, L. A., ANDERSON, T. & KOHN, K. W. (1979) DNA-protein and DNA interstrand cross linking by cis- and trans-Platinum (II) Diammine dichloride in L1210 mouse leukaemia cells and relation to cytotoxicity. *Cancer Res.*, **39**, 365.